# Stat3 and Stat5 govern IL-10 expression in T cells through *trans*-activation and epigenetic remodelling in health and disease

**DOI:** 10.1186/2194-7791-1-S1-A17

**Published:** 2014-09-11

**Authors:** CM Hedrich, T Rauen, JC Crispin, GC Tsokos

**Affiliations:** Klinik und Poliklinik für Kinder- und Jugendmedizin, Universitätsklinikum Carl Gustav Carus, TU Dresden, Germany; Rheumatology, Beth Israel Deaconess Medical Center, Boston, MA USA

IL-10 is an immune-regulatory cytokine that plays a central role during innate and adaptive immune responses. T cells are a major source of IL-10. The molecular mechanisms governing IL-10 expression remain only partially understood.

The autoimmune disorder systemic lupus erythematosus (SLE) is characterized by autoantibody production, immune complex formation, and altered cytokine expression. IL-10 is elevated in the serum and tissues of SLE patients. Next to its anti-inflammatory capacities, IL-10 promotes the differentiation, survival, and activity of B cells. Thus, IL-10 contributes to autoantibody production and tissue damage in SLE.

The molecular events contributing to the increased expression of IL-10 in SLE patients remain to be determined. We aimed to determine molecular mechanisms controlling *IL10* in health and disease.

In T cells, DNA methylation of the *IL10* promoter and the 4^th^ intron governs the recruitment of Stat transcription factors. Both Stat3 and Stat5, which are recruited to the 5’ proximal promoter and the 4th intron, regulate IL-10. Stat3 and Stat5 mediate both *trans*-activation and epigenetic remodelling through their interaction with the histone acetyltransferase p300. In T cells from SLE patients, activation of Stat3 is increased, resulting in a replacement of Stat5, subsequently promoting IL-10 expression.

Understanding the molecular events contributing to cytokine deregulation in SLE will offer new therapeutic options. Correcting the imbalanced activation of Stat transcription factors may be a promising candidate in the search for novel therapeutic approaches in SLE.

